# Supplementary material for: Laboratory Evaluation of Low-Cost Optical Particle Counters for Environmental and Occupational Exposures
Source: Sensors (Basel). 2021 Jun 17;21(12):4146. doi: 10.3390/s21124146 (PMC8233711; doi:10.3390/s21124146)
Supplement: Supplementary file 1 [file sensors-21-04146-s001.zip › sensors-1253009-supplementary.pdf]

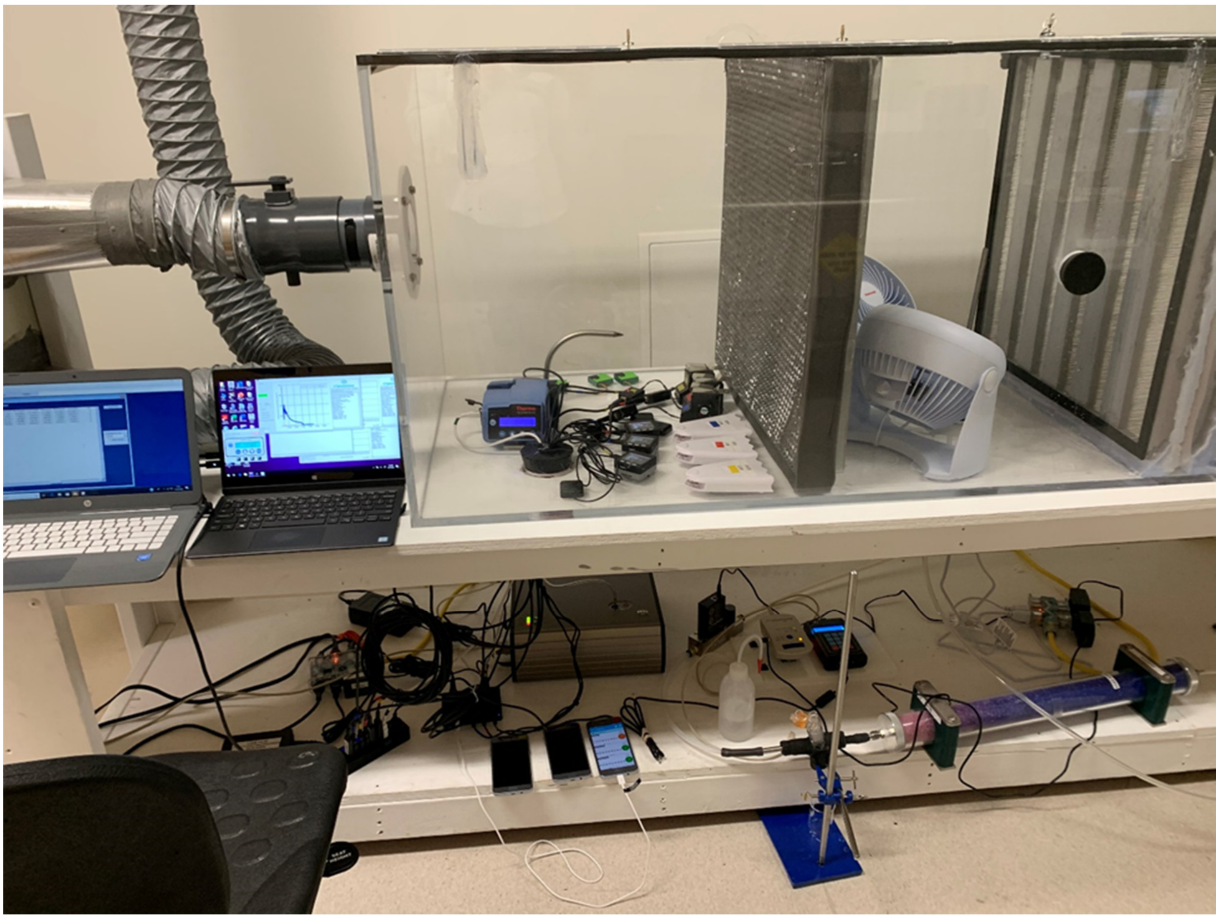

Figure S1: Photo of the experimental setup inside the Aerosol Research Laboratory at East Carolina University.

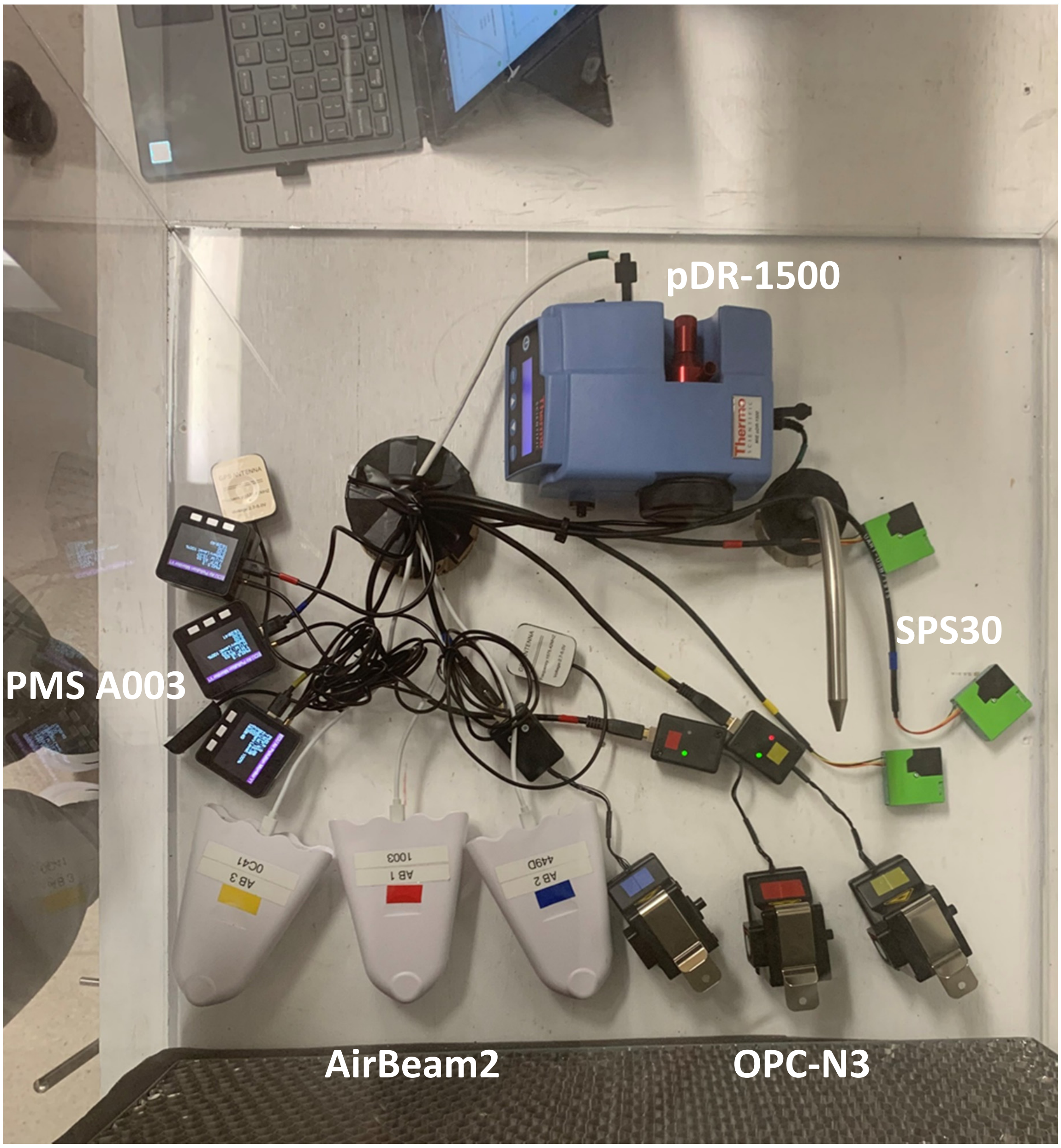

Figure S2: Photo of the low-cost sensors inside the sampling zone of the chamber.

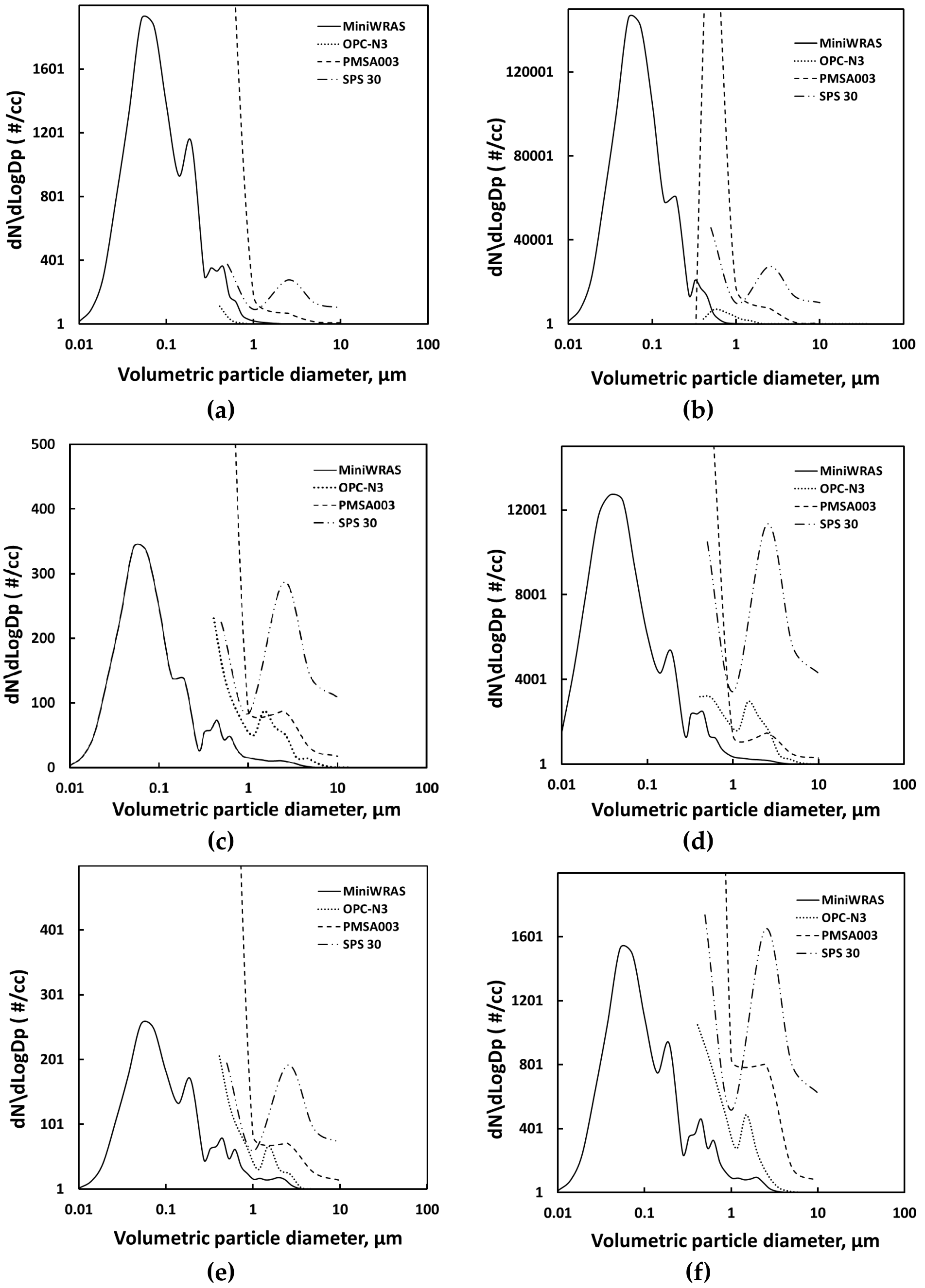

Figure S3: Particle number concentration for low-cost sensors and the reference instrument for (**a, b**) salt, (**c, d**) ARD, and (**e, f**) PAO-4 oil (environmental and occupational settings, respectively).
